# Revisiting AI Interpretability in Precision Oncology: Why Predictive Accuracy Does Not Ensure Stable Feature Importance

**DOI:** 10.3390/cancers18040593

**Published:** 2026-02-11

**Authors:** Souichi Oka, Yoshiyasu Takefuji

**Affiliations:** 1 Science Park Corporation, 3-24-9 Iriya-Nishi, Zama 252-0029, Japan; 2 Faculty of Data Science, Musashino University, 3-3-3 Ariake, Koto-ku, Tokyo 135-8181, Japan; takefuji@keio.jp

**Keywords:** artificial intelligence, predictive accuracy, interpretability, feature importance, feature ranking consistency, multi-omics, explainable AI

## Abstract

Background: Artificial intelligence (AI) is becoming important in oncology, supporting risk prediction, treatment planning, and biomarker discovery. However, current evaluation practices often assume that high predictive accuracy implies reliable interpretation—a misconception that may undermine reproducibility and clinical decision-making. This study aims to reassess interpretability by introducing feature ranking order consistency as a stability-focused metric to evaluate how model explanations respond to minimal input perturbations. Methods: Using The Cancer Genome Atlas (TCGA) breast cancer multi-omics dataset, we compared supervised models—Linear Regression, Least Absolute Shrinkage and Selection Operator (LASSO), Random Forest, and Extreme Gradient Boosting (XGBoost)—with unsupervised and statistical methods, including Principal Component Analysis (PCA), Highly Variable Gene Selection, and Spearman’s rank correlation. Each method produced a Top 20 feature ranking, and stability was assessed by testing whether rankings remained consistent after removing the top-ranked feature. Predictive performance was evaluated using a Random Forest classifier with stratified 10-fold cross-validation. Results: Supervised models exhibited unstable feature importance rankings even under minimal perturbations (<0.1% feature removal), suggesting that high predictive accuracy may obscure fragile or misleading explanations. In contrast, Highly Variable Gene Selection and Spearman’s correlation consistently produced stable, biologically coherent feature sets and maintained competitive predictive performance. Conclusions: Interpretive instability is a major limitation of many machine learning models in oncology. Incorporating stability-based criteria—such as feature ranking consistency—into evaluation frameworks is essential for ensuring reproducible, trustworthy, and clinically actionable AI. As AI adoption accelerates, prioritizing interpretability alongside accuracy is critical for responsible deployment in precision oncology.

## Simple Summary

Artificial intelligence (AI) is becoming a powerful tool in cancer research, helping researchers and clinicians predict patient outcomes and identify important biological markers. However, many AI models can appear highly accurate while still giving unstable or unreliable explanations about which factors are truly critical. This study evaluates the consistency and reliability of different AI methods in the analysis of complex breast cancer data. We found that some popular machine learning models change their explanations dramatically with only tiny changes to the input, raising concerns about their reliability. In contrast, simpler data-driven approaches identified important features more consistently and still achieved superior predictive performance. These findings highlight the importance of evaluating not only how accurate an AI model is, but also how stable and transparent its reasoning is. Improving the stability of AI explanations can support the development of safer, more dependable tools for understanding cancer and guiding future decisions.

## 1. Introduction

Artificial intelligence (AI) is increasingly influencing oncology practice worldwide, offering opportunities to improve diagnosis, treatment planning, and patient outcomes. These technological advancements are particularly significant in light of the rising global cancer burden. Cancer remains one of the most pressing global public health challenges, with the number of new cases and deaths continuing to rise worldwide. Recent analyses published in *Cancers* have emphasized the accelerating increase in global cancer incidence and mortality across regions [1]. In this context, AI-driven tools are expected to improve efficiency and accuracy, but their rapid adoption raises critical concerns about trustworthy interpretation—whether model explanations are sufficiently stable, transparent, and reliable to support patient safety and evidence-based policy.

The growing importance of AI in oncology is also reflected in *Cancers*, which now hosts multiple Special Issues and topical collections dedicated to AI- and machine-learning-based approaches in cancer research. However, as this trend accelerates, it is essential to proceed with caution. In oncology, AI can serve two distinct roles: predicting “who might develop cancer” and explaining “why cancer occurs.” Current research often conflates these roles, assuming that high predictive accuracy implies reliable causal interpretation—a misconception that has been widely criticized in influential studies [2,3].

Many commonly used supervised learning models achieve impressive predictive performance while producing fragile and inconsistent interpretive outputs, risking the misidentification of truly important factors [4,5,6]. The underlying problem stems from structural biases: linear models often oversimplify relationships [7,8,9]; dimensionality-reduction approaches can discard information critical for complex biological systems [10,11,12,13,14,15]; tree-based ensembles tend to overweight features selected early in the splitting process, skewing importance scores [16,17,18,19,20,21,22,23,24]; and deep learning architectures are prone to overfitting and reliance on spurious patterns [25]. These design constraints mean that high accuracy can mask interpretive fragility, because most models capture superficial correlations rather than underlying causal mechanisms.

Most prior studies present a feature-importance ranking obtained from model output without directly evaluating whether those rankings are stable across small perturbations of the data. Yet if a ranking changes substantially in response to even minimal perturbations, it suggests that the model may be capturing noise-driven or dataset-specific patterns rather than robust biological signals. Assessing how feature rankings respond to such perturbations therefore provides a practical and informative measure of their reliability. In this study, we focus on this perspective to determine whether feature-importance rankings genuinely reflect stable and biologically meaningful relationships, or whether they are vulnerable to model-dependent variability. These considerations underscore the need for feature-selection approaches that are inherently more stable.

To overcome these limitations, data-centric interpretability provides a promising alternative. Unsupervised techniques (e.g., Highly Variable Gene Selection) and non-parametric statistical filtering (e.g., Spearman’s rank correlation) identify features based on intrinsic data structure rather than model-driven heuristics [26,27,28,29]. By minimizing reliance on prior assumptions and extensive algorithmic tuning, these approaches reduce the risk of overfitting and consistently yield more stable, reproducible explanations in high-dimensional and complex settings. Highly Variable Gene Selection is a simple variance-based filtering strategy that isolates features exhibiting genuine biological variability while suppressing technical noise. Although routinely used in many transcriptomic workflows—including modern single-cell pipelines—its application to multi-omics feature-selection problems has been comparatively limited.

A broader examination of the recent biomedical machine-learning literature reveals a pronounced methodological imbalance: supervised learning models overwhelmingly dominate mainstream research, whereas data-centric feature-selection strategies are employed only marginally. To contextualize this pattern, we conducted a broad exploratory search utilizing standard keyword-matching functions available across major scholarly publishing platforms, aiming to capture representative methodological trends rather than perform a formal systematic review. Across this extensive landscape, Highly Variable Gene Selection and Spearman’s rank correlation—both of which consistently demonstrate high stability and interpretability in high-dimensional settings—appeared only infrequently. This underrepresentation indicates that robust and reproducible feature-selection strategies remain largely overlooked, overshadowed by accuracy-driven, model-dependent techniques that may offer limited interpretive reliability.

Given the growing interest in data-driven oncology and the focus of this Special Issue on advanced computational methods in cancer research, this study contributes a timely evaluation of interpretability-centered AI approaches that address key limitations in current predictive modeling practices. We aim to demonstrate the comparative advantages of underutilized approaches—such as unsupervised techniques and statistical filtering—over prevailing supervised learning models with respect to interpretive robustness. To accomplish this objective, we conducted a comparative analysis using The Cancer Genome Atlas (TCGA) breast cancer multi-omics dataset [30]. Our findings indicate that widely trusted supervised AI methods, despite their high predictive accuracy, exhibit striking interpretive fragility and frequently misidentify key factors under minimal perturbations. Conversely, unsupervised techniques and statistical filtering consistently deliver greater stability and, in several cases, superior predictive performance.

These findings challenge prevailing assumptions and underscore the need for evaluation standards that prioritize interpretive robustness alongside accuracy. From a cancer policy perspective, this distinction is critical because decisions on screening strategies, resource allocation, and clinical guidelines depend on AI-based decision-support models that must provide trustworthy explanations. Determining which AI approaches are suitable for interpretation—not merely prediction—should be considered a cornerstone of regulatory frameworks and evidence-based policy, supporting patient safety and equitable care.

## 2. Materials and Methods

### 2.1. Multi-Omics Cancer Dataset

As a widely used and well-curated resource for genomics and translational oncology research, we utilized the breast cancer multi-omics dataset from The Cancer Genome Atlas (TCGA) [30]. All samples were collected under the standardized data-generation protocols of the TCGA consortium. This dataset comprises samples from 705 patients, including two major histological subtypes: Invasive Ductal Carcinoma (IDC) and Invasive Lobular Carcinoma (ILC). At the time of the last follow-up, 611 (86.7%) patients were alive and 94 (13.3%) were deceased. No class reweighting, resampling, or threshold optimization was applied, in order to isolate and evaluate the intrinsic information content of the selected feature sets under strictly identical downstream evaluation conditions. The clinical outcome of interest, survival status, is recorded under the column name “vital.status,” and was used as the target outcome for predictive analyses. The dataset includes 1936 features spanning multiple molecular layers, such as gene mutations, mRNA expression profiles, and additional omics modalities, providing a comprehensive representation of the biological landscape associated with patient outcomes. All data used in this study were publicly available and de-identified; therefore, institutional review board (IRB) approval was not required. Missing values were imputed with zeros, and categorical variables were one-hot encoded. No additional scaling or normalization was applied.

### 2.2. Feature Importance and Selection Methods

We compared seven representative methods, grouped into three distinct categories, to compute feature importance and generate rankings. These methods were carefully selected to reflect widely used approaches in modern data science, each grounded in a distinct theoretical framework.

First, from supervised machine learning, we included both linear models—Linear Regression and LASSO—and nonlinear ensemble models—Random Forest and XGBoost—which are currently mainstream due to their strong predictive performance. These models were included to examine whether high predictive accuracy is associated with stable feature importance rankings.

Second, from unsupervised machine learning, we selected Principal Component Analysis (PCA), a classical linear dimensionality reduction technique; and Highly Variable Gene Selection, which is commonly applied in multi-omics analyses.

Third, from statistical filtering, we included Spearman’s rank correlation, a fundamental non-parametric statistical method, serving as a robust baseline independent of model-specific assumptions.

This selection enables a systematic investigation of how methodological characteristics—such as supervised vs. unsupervised, linear vs. nonlinear, and parametric vs. non-parametric approaches—influence the stability of feature rankings in practical applications. To ensure fairness and reproducibility, all simulations were conducted using default parameters provided by widely used Python libraries across analyses in this study as implemented. All analyses were conducted using Python 3.13.7, with scikit-learn 1.8.0, XGBoost 3.0.4, NumPy 2.3.2, and pandas 2.3.2.

Feature importance was computed as follows. For Linear Regression and LASSO, importance was defined as the absolute value of the fitted regression coefficients. For Random Forest, we used the default mean decrease in impurity (Gini importance) as implemented in scikit-learn. For XGBoost, feature importance was taken from the model’s default importance measure, which is gain-based. For PCA, features were ranked according to the sum of the absolute values of their component loadings across all components. For Highly Variable Gene Selection, importance was defined by feature-wise variance, and for Spearman’s method, by the absolute Spearman rank correlation with the outcome variable.

### 2.3. Evaluation Framework

#### 2.3.1. Feature Ranking Order Consistency

**Figure 1:**
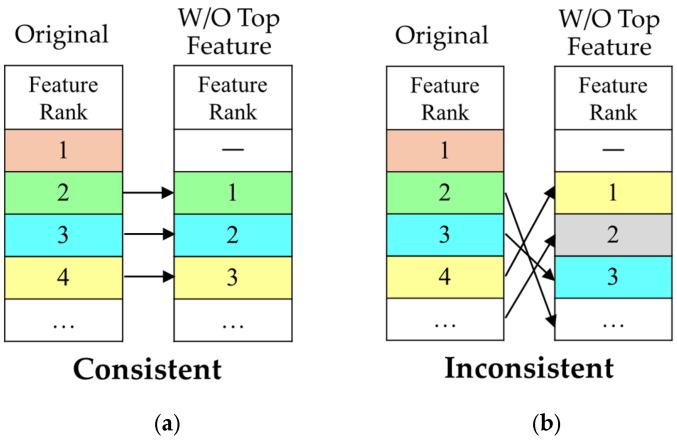
Evaluation framework for ranking consistency: (**a**) shows a “Consistent” outcome, where the remaining feature set and order are unchanged after removing the top-ranked feature; (**b**) illustrates an “Inconsistent” outcome, where the feature set and order are altered following the perturbation. Colors denote feature identity, with the same color indicating the same feature across panels.

To evaluate the stability of feature ranking, we applied a controlled perturbation framework as shown in Figure 1. For each method, an initial Top 20 feature ranking was obtained from the full dataset based on feature importance scores. Then, the highest-ranked feature was removed, and the method was reapplied to the dataset without the top feature to generate a new ranking.

The criterion for evaluating ranking order consistency was defined rigorously. A method was classified as “Consistent” only if the set of features ranked 2nd to 20th in the initial ranking exactly matched the set of features ranked 1st to 19th in the perturbed ranking, with their relative order preserved. As shown in Figure 1a, this corresponds to a uniform rank shift, where the feature initially ranked 2nd becomes 1st, the 3rd becomes 2nd, and so forth. If any feature differed between the two sets or if the relative order was altered, the method was classified as “Inconsistent.” Figure 1b illustrates an example where the original ranking structure collapses, resulting in a substantially reordered feature list. This strict definition enables a direct assessment of robustness to minimal input perturbations.

Prior work has already shown that subset-based stability metrics—such as the Jaccard or Kuncheva indices—are fundamentally inadequate for interpretability, as they measure only set overlap and remain blind to substantial reorderings in feature importance [31]. Hamer and Dupont demonstrated that these metrics can even label models as “stable” while their internal importance structure shifts markedly [32]. Gyawali et al. similarly showed that stochastic variation in modern models can induce large, meaningful ranking fluctuations that ensemble methods tend to obscure [25]. These findings make clear that existing measures have already been shown to miss the specific instability that matters most: whether the explanatory hierarchy itself survives minimal perturbation. This gap motivates our stricter criterion. By requiring complete preservation of the relative ordering among remaining features, it directly reveals collapses in interpretive structure that overlap-based or smoothed metrics are structurally incapable of detecting.

To avoid any randomness when multiple features shared the same importance score (i.e., ties), we used a stable sorting algorithm. In such ties, the relative order was determined deterministically by the original column order of the features, preventing any random reordering and ensuring fully reproducible rankings.

#### 2.3.2. Predictive Accuracy

To assess the practical utility and information content of the feature subsets selected by each method, we evaluated their predictive performance using a common downstream classifier. Specifically, a Random Forest classifier with 100 trees was employed, following the default settings of the scikit-learn library. For each feature selection method, the top 20 ranked features were used as input variables. Model performance was evaluated via stratified 10-fold cross-validation. This evaluation was repeated over 10 different random seeds. For the positive (deceased) class, the mean accuracy, mean sensitivity and binary F1 score were reported across folds. To avoid information leakage, feature selection was recomputed within each training fold during cross-validation. No explicit batch-effect correction was applied, as the analysis focused on comparative stability under identical preprocessing.

A fixed Random Forest classifier was used as the downstream evaluator to ensure a consistent, fair, and method-agnostic assessment of the information content of the selected feature sets. While alternative downstream classifiers could be considered, using a single evaluator was a deliberate design choice to isolate differences attributable to feature selection rather than to the choice of classifier.

No generative artificial intelligence (GenAI) tools were used for any purpose in the preparation of this manuscript, including text generation, data processing, figure creation, or any form of editing. All components of this work were produced entirely through standard scientific practices without the assistance of GenAI.

### 2.4. Parameter Settings

To ensure methodological consistency and fairness across all evaluated models, widely used and minimally adjusted parameter settings were applied throughout the analyses. Random Forest was implemented with 100 estimators and class-weight balancing to accommodate the substantial class imbalance in survival status, a configuration that provides stable performance without model-specific tuning. LASSO was used with a mild regularization strength (alpha = 0.01) and an increased iteration limit to ensure numerical convergence while avoiding excessive sparsity. Linear regression employed default settings, which is appropriate given that regression coefficients were used solely to derive feature-importance magnitudes. XGBoost was run with its standard binary logistic objective and default hyperparameters, reflecting the intention to evaluate feature-importance behavior without introducing method-specific optimization. For unsupervised and statistical baselines, Highly Variable Gene Selection was computed from feature-wise variance, Spearman’s method from absolute rank correlation with the outcome, and PCA from the sum of absolute component loadings. All feature-selection procedures and comparative evaluations were conducted under identical preprocessing and cross-validation conditions, enabling differences in performance or ranking stability to be attributed to the methodological nature of each approach rather than to subtle disparities in parameter configuration.

## 3. Results

Table 1 presents the feature importance rankings for the multi-omics dataset using supervised machine learning models. Linear Regression, LASSO, Random Forest, and XGBoost exhibited significant inconsistencies between the initial and perturbed rankings.

Table 2 shows the rankings derived from unsupervised learning and statistical filtering methods. PCA produced inconsistent rankings, whereas Highly Variable Gene Selection and Spearman’s rho demonstrated complete consistency between the initial and perturbed rankings.

We also examined whether feature-selection results were influenced by random seeds. Among the evaluated methods, only Random Forest and XGBoost include stochastic components and could theoretically exhibit seed-dependent variation, whereas Linear Regression, LASSO, PCA, Highly Variable Gene Selection and Spearman’s rho are fully deterministic under fixed data and hyperparameters. In line with this distinction, Random Forest displayed modest seed-dependent variability in feature rankings, whereas XGBoost yielded identical rankings across all tested seeds, consistent with the deterministic behavior of its greedy split-selection mechanism in the absence of subsampling and additional randomness.

Table 3 summarizes the predictive accuracy, sensitivity, F1 score and ranking consistency for each method on the multi-omics dataset. Highly Variable Gene Selection achieved the highest accuracy (0.8862 ± 0.0025), sensitivity (0.2255 ± 0.0150) and F1 score (0.3314 ± 0.0218), being classified as “Consistent”. The ± value represents the standard deviation across the folds in stratified 10-fold cross-validation, reflecting the variability in model performance. These values reflect the average performance of the downstream Random Forest evaluator across 10 random seeds.

Although most methods achieved relatively high predictive performance, the rankings produced by Linear Regression, LASSO, Random Forest, XGBoost, and PCA were all classified as “Inconsistent”. These findings further support the conclusion that predictive accuracy and ranking consistency are not necessarily correlated.

## 4. Discussion

### 4.1. Interpretation of Feature Selection

In the multi-omics cancer data setting, we conducted a feature importance analysis based on the TCGA breast cancer cohort, which comprises two major histological subtypes: Invasive Lobular Carcinoma (ILC) and Invasive Ductal Carcinoma (IDC). The prediction target was vital status (survival outcome), and the input features spanned multiple molecular layers, including DNA sequencing, RNA expression, and RPPA-based protein expression measurements.

This analysis highlighted several molecular drivers strongly associated with survival prognosis, such as activation of the PTEN-AKT signaling pathway and mutations in TBX3 and FOXA1, findings that are consistent with established breast cancer biology. These results underscore the critical importance of identifying molecular markers that are genuinely associated with patient survival in multi-omics data, a task with direct implications for human health. Regarding the central theme of this study—feature importance consistency—Highly Variable Gene Selection and Spearman’s rank correlation demonstrated notably high stability across repeated analyses, supporting their suitability for robust and reproducible feature prioritization.

Highly Variable Gene Selection showed a clear advantage in identifying clinically interpretable and stable feature sets, achieving perfect ranking consistency. Notably, strong biological signals such as rs_ADH1B—a known breast cancer risk factor related to alcohol metabolism—may also appear among top-ranked features even when unstable methods like linear regression are applied [33]. Here, the prefix “rs” stands for Reference SNP cluster ID, a standardized identifier used in the dbSNP database maintained by NCBI. Each rsID corresponds to a specific single nucleotide polymorphism (SNP)—a common type of genetic variation among individuals. In essence, the true strength of Highly Variable Gene Selection lies in its ability to consistently identify not only such prominent markers but also a diverse and biologically coherent set of features spanning multiple molecular domains. Specifically, Highly Variable Gene Selection consistently prioritized known markers such as:

- rs_SCGB2A2 and rs_TFF1: estrogen response [34,35];
- rs_GSTM1: detoxification enzyme [36];
- rs_ADIPOQ: adiponectin, linking obesity and prognosis [37];
- rs_PIP: tumor marker [38].

This ability to construct a reliable panel of markers reflecting hormonal response, metabolism, obesity-related factors, and tumor biology is a key methodological advantage of Highly Variable Gene Selection.

Similarly, Spearman’s rho, a non-parametric measure of rank association, demonstrated strong stability in identifying clinically meaningful markers. For example, rs_PIK3C2G, which is involved in cell proliferation signaling, represents a biologically salient feature that may occasionally appear among top-ranked markers even when unstable methods like LASSO are applied [39]. In contrast, Spearman’s rho consistently prioritized this marker together with others capturing diverse biological processes such as:

- rs_CD36: lipid metabolism and metastasis [40];
- rs_PCOLCE2: tumor microenvironment [41];
- rs_C6: immune response via complement system [42];
- rs_TUSC5: candidate tumor suppressor gene [43].

These results indicate that Spearman’s rho, despite its simplicity, offers substantial practical utility for the reliable identification of biomarkers across a broad range of biological contexts, spanning cancer cell–intrinsic properties, immune responses, and microenvironmental factors.

By comparison, several commonly used methods exhibited limited ranking stability, a limitation that is particularly pronounced in high-dimensional and heterogeneous omics data. Although these approaches often contributed to predictive performance, the clinical interpretability of their top-ranked features remains limited and becomes apparent when examining specific examples such as:

- rs_CHIT1 (selected by PCA): encodes chitinase produced by macrophages; may relate to inflammation but not central to breast cancer prognosis [44];
- rs_ABCA12: lipid transporter in skin cells; possibly reflects secondary phenomena [45];
- rs_KIAA0408 (Random Forest): gene with poorly understood function; statistically effective but biologically obscure;
- rs_PLIN1 (XGBoost): encodes a protein coating lipid droplet in adipocytes; likely reflects surrounding adipose tissue rather than tumor biology [46].

These examples illustrate that in the pursuit of maximizing predictive performance, models may fail to select biologically central or stable features. This highlights a serious trade-off between predictive accuracy and interpretability/stability, especially in biomarker discovery for clinical applications.

### 4.2. Reconsidering Analogies in Feature Importance Analysis

Through empirical simulation, this study quantitatively demonstrates that high predictive accuracy does not necessarily imply reliable feature importance rankings. This observation is further supported by extensive evidence from the biomedical literature. Together, these results underscore that ranking consistency is a crucial metric for evaluating the interpretability of machine learning models, beyond predictive performance alone. Notably, removing just a single feature—1 out of 1936 (0.052%)—was sufficient to reveal substantial instability in feature importance rankings for high-performing supervised models such as Random Forest and XGBoost. This instability highlights that these models often rely on non-robust, data-specific correlations that are vulnerable to even minimal perturbations.

A common defense of this instability invokes the analogy: “When a star player is missing from a sports team, the strategy changes and the importance of other players shift.” While intuitive, this comparison is misleading because machine learning models do not adapt strategically in the way human teams do; instead, they reconfigure feature weights through stochastic processes and greedy optimization, without explicit causal reasoning. As a result, the apparent “strategy shift” in AI models reflects a non-deliberate reconfiguration rather than a logical or goal-directed adaptation. Misinterpreting such behavior as human-like decision-making can distort scientific conclusions and compromise policy decisions. These findings highlight the need to prioritize interpretive stability—alongside accuracy—when integrating AI into cancer policy frameworks.

### 4.3. Robustness of Ranking Stability Under Feature Selection Variations

These findings indicate that, when integrating AI into cancer policy frameworks, interpretive stability should be treated as a criterion at least as important as predictive accuracy. From this standpoint, our perturbation analysis is deliberately designed to probe model behavior under minimal change, because instability revealed at this level is sufficient to assess the robustness of feature importance rankings.

While this aspect is not central to the proposed framework, we conducted a confirmatory check and verified that removing larger subsets of top-ranked features (up to Top-10) does not alter the qualitative conclusions. However, once the ranking structure collapses under a minimal perturbation—namely, the removal of a single feature—it follows as a matter of logical necessity that removing larger feature sets cannot restore stability but can only exacerbate the observed instability. In contrast, the ranking invariance observed for Spearman’s rank correlation and Highly Variable Gene Selection is not an empirical coincidence, but an inevitable consequence of their methodological design: each feature is evaluated independently using univariate criteria, under which the relative ordering among remaining features cannot change regardless of which, or how many, features are removed. For these reasons, such additional results are informationally redundant and are therefore omitted.

Although the absolute F1 scores are moderate, this outcome is not unexpected given the pronounced class imbalance in the survival prediction task (approximately 13% deceased). Under such conditions, a binary F1 score of around 0.33, as achieved by the best-performing method (Highly Variable Gene Selection), indicates that the model captures a non-trivial fraction of the minority class beyond a majority-class or random baseline.

To better understand these results, we next compare the evaluated methods across multiple performance metrics. As shown in Table 3, the evaluated methods achieved broadly comparable overall accuracy, whereas sensitivity and F1 score exhibited greater dispersion across methods. Highly Variable Gene Selection ranked first across all three metrics; however, its advantage in accuracy over other approaches was marginal, while the separation in sensitivity and F1 score was more pronounced.

In oncology applications, prediction targets are intrinsically tied to patient survival, and the consequences of misclassification are therefore asymmetric. In particular, failing to identify patients at elevated risk of death carries more serious clinical implications than incorrectly classifying low-risk patients as high risk. Under such class-imbalanced conditions, accuracy is largely driven by correct classification of the majority class and consequently becomes relatively insensitive to differences in detecting high-risk cases. Sensitivity, by contrast, directly captures the ability to identify deceased patients and is thus more closely aligned with the clinical objective of risk stratification. In cancer outcome prediction, absolute differences in sensitivity are consequential, because the target of prediction is patient survival. For example, Highly Variable Gene Selection achieved a sensitivity of 0.2255, compared with 0.2044 for Random Forest, corresponding to an absolute difference of approximately 2%. Such discrepancies are particularly relevant in this setting, where false negatives correspond to high-risk patients whose elevated mortality risk remains unrecognized.

To assess whether our conclusions depend on the number of selected top-ranked features, we conducted an additional sensitivity analysis by systematically varying the feature set size. Across a moderate range (Top 10 to Top 25 features), the qualitative conclusions remained unchanged, indicating that the observed differences in ranking stability are robust to this choice. When larger feature sets (≥30 features) were considered, sensitivity for the minority class reached a clear plateau, suggesting that the essential prognostic features had already been captured. Beyond this point, further increases in overall accuracy were driven primarily by gains in specificity for the majority class, rather than improved detection of high-risk cases. Accordingly, selecting the top 20 features provides a balanced and informative setting that captures the core prognostic signal while avoiding dilution by redundant or weakly informative features.

### 4.4. Limitations

To preserve methodological consistency, our analysis focused on models that natively provide explicit feature-importance scores, including linear regression, LASSO, random forest, and XGBoost. Support Vector Machines (SVMs) were not included, because the formulations commonly used in high-dimensional biomedical research are kernel SVMs, which do not yield feature-level importance. Although linear SVMs possess coefficients, we did not regard them as native importance measures within our framework, as such coefficients do not generalize to kernelized decision boundaries and thus fall outside our definition of intrinsic feature ranking. PCA was included solely as an unsupervised baseline to evaluate its behavior under minimal perturbation, and its loadings were not interpreted as supervised feature-importance scores.

Parametric deep learning approaches were likewise excluded because they rely on high-dimensional latent representations rather than explicit feature weights. Their interpretability necessarily depends on post hoc attribution techniques (e.g., Integrated Gradients or gradient-based saliency), which are fundamentally different from the intrinsic ranking mechanisms examined here. Incorporating such models would therefore shift the analysis away from our primary objective: assessing the stability of explicit, model-native feature-importance rankings.

A further limitation concerns the outcome definition used in this study. Consistent with standard TCGA benchmark settings, vital status was analyzed as a binary outcome; consequently, time-to-event information was not considered, which restricts the survival-specific interpretation of the results.

## 5. Conclusions

AI is increasingly shaping oncology, yet our findings demonstrate that high predictive accuracy does not guarantee reliable interpretive value. Using TCGA breast cancer multi-omics data, we showed that widely used supervised and dimensionality-reduction methods—including Linear Regression, LASSO, Random Forest, XGBoost, and PCA—often produce unstable feature importance rankings under minimal perturbations. Such fragility presents risks when these models are used to inform decisions in policy-relevant domains such as screening prioritization, resource allocation, and clinical guideline development. In contrast, Highly Variable Gene Selection and Spearman’s rank correlation delivered stable, biologically coherent, and reproducible feature rankings while maintaining strong predictive performance, demonstrating that interpretive stability and accuracy can indeed coexist.

These findings underscore the need for oncology and precision-medicine frameworks to prioritize interpretive robustness—not merely accuracy—when deploying AI models for therapeutic response prediction, biomarker discovery, and data-driven clinical decision support. As emphasized in this Special Issue’s focus on explainable, trustworthy, and clinically actionable AI, establishing evaluation standards that incorporate stability criteria is essential for ensuring reproducibility, patient safety, and equitable care. Ultimately, the responsible integration of AI into oncology requires methodological choices guided by empirical rigor rather than algorithmic novelty, forming the foundation for evidence-based regulation and ethically sound clinical decision-making.

## Figures and Tables

**Table 1:** Feature rankings from supervised machine learning models.

Linear Regression		LASSO		Random Forest		XGBoost	
Ranking (Original Set)	Ranking (W/O Top 1)	Ranking (Original Set)	Ranking (W/O Top 1)	Ranking (Original Set)	Ranking (W/O Top 1)	Ranking (Original Set)	Ranking (W/O Top 1)
rs_PLA2G2D	―	cn_TNFRSF11B	―	rs_KIAA0408	―	rs_ACVR1C	―
rs_ACVR1C	rs_ACVR1C	cn_ABCC6	cn_ABCC6	rs_LOC283392	rs_IL33	rs_PLIN1	rs_PLIN1
rs_ADH1B	rs_ADH1B	cn_BRDT	cn_BRDT	rs_LEP	rs_ADH1B	rs_TUSC5	rs_MMRN1
rs_C6	rs_C6	cn_IDO1	cn_IDO1	rs_MMRN1	rs_APOB	rs_IGDCC3	rs_SHISA2
rs_ACTG2	rs_GRIA4	rs_AR	rs_AR	rs_POF1B	rs_LOC283392	rs_SPDYC	rs_SYT13
rs_GRIA4	rs_ACTG2	rs_IL33	rs_IL33	rs_FIBCD1	rs_KCNIP2	rs_MAPK4	rs_MAGEA4
rs_HEPN1	rs_HEPN1	rs_ANO3	cn_COL14A1	rs_APOB	rs_POF1B	rs_KCNJ3	rs_APOB
cn_ANK1	rs_ORM2	rs_PCOLCE2	cn_COL10A1	rs_ABCA8	rs_FIBCD1	rs_ADCY5	rs_TSPAN8
rs_ORM2	cn_COCH	rs_ORM2	rs_PCOLCE2	rs_NPY2R	rs_SLC19A3	rs_POF1B	rs_SPDYC
cn_COCH	rs_KRT14	rs_MMP12	rs_ANO3	rs_SLC19A3	rs_PLIN1	rs_GABBR2	rs_TUSC5
rs_KRT14	rs_SLC13A2	rs_AKR1B10	rs_ORM2	rs_HEPN1	rs_ACVR1C	rs_KRT13	rs_DACH1
cn_TRIM58	pp_Akt.pT308	cn_COL10A1	rs_MMP12	rs_GLYAT	rs_FXYD1	rs_ORM1	mu_ITPR2
rs_SLC13A2	rs_TMEM45B	cn_CDC20	rs_AKR1B10	rs_ACVR1C	rs_ABCA8	pp_Akt	rs_FAM5C
pp_Akt.pT308	pp_DJ.1	rs_C6	rs_TPSD1	rs_PCOLCE2	rs_TNMD	rs_SHISA2	cn_CGA
rs_TMEM45B	cn_TRIM58	rs_TPSD1	rs_C6	rs_CXCL2	rs_FABP4	rs_HOXC10	rs_C10orf82
rs_PART1	cn_NPTX1	rs_APOB	rs_APOB	rs_C14orf180	rs_NEK10	rs_CYP4F22	rs_MYT1
cn_TNFRSF11B	rs_PART1	rs_CYP2A7	rs_HEPACAM	rs_OXTR	rs_TMEM132C	rs_PPP2R2C	pp_Smad3
pp_DJ.1	cn_ANK1	rs_PPP1R1B	rs_PIK3C2G	rs_KCNIP2	rs_ADH1C	pp_Bad.pS112	rs_TFAP2B
rs_CXCR2P1	cn_TNFRSF11B	rs_PIK3C2G	cn_CDC20	rs_HEPACAM	rs_AQP7	rs_FIBCD1	rs_CHRM1
rs_C4BPA	rs_MS4A1	rs_HEPACAM	rs_CYP2A7	rs_IL33	rs_MUC15	rs_CAPN8	pp_p90RSK

This table presents the top feature rankings generated by the four supervised machine learning models on the multi-omics cancer dataset. Feature names are prefixed to indicate their data type: rs_ for Reference SNP cluster ID (a standardized identifier for single nucleotide polymorphisms), cn_ for copy number variations, mu_ for mutations, and pp_ for phosphorylated proteins. All four models exhibit significant ranking inconsistency, as the feature order in the ‘W/O Top 1’ column differs substantially from that of the ‘Original set’.

**Table 2:** Feature rankings from unsupervised machine learning models and statistical filtering.

PCA		Highly Variable Gene Selection		Spearman’s Rho	
Ranking (Original Set)	Ranking (W/O Top 1)	Ranking (Original Set)	Ranking (W/O Top 1)	Ranking (Original Set)	Ranking (W/O Top 1)
rs_CHIT1	―	rs_CLEC3A	―	rs_PIK3C2G	―
rs_ABCA12	rs_HGD	rs_CPB1	rs_CPB1	rs_ANO3	rs_ANO3
rs_RIMS2	rs_NELL2	rs_SCGB2A2	rs_SCGB2A2	rs_PCOLCE2	rs_PCOLCE2
rs_SLC9A2	rs_ABCA12	rs_GSTM1	rs_GSTM1	rs_CD36	rs_CD36
rs_CRYM	rs_CYP4F11	rs_MUCL1	rs_MUCL1	rs_CIDEC	rs_CIDEC
rs_IYD	rs_ATRNL1	rs_TFF1	rs_TFF1	rs_PCK1	rs_PCK1
rs_NELL2	rs_SERPINA5	rs_SCGB1D2	rs_SCGB1D2	rs_AQP7	rs_AQP7
rs_CEACAM5	rs_KRT15	rs_ADH1B	rs_ADH1B	rs_TUSC5	rs_TUSC5
rs_C16orf89	rs_CRYM	rs_ADIPOQ	rs_ADIPOQ	rs_C14orf180	rs_C14orf180
rs_KRT4	rs_KRT4	rs_PIP	rs_PIP	rs_ADH1B	rs_ADH1B
rs_C2orf54	rs_SCGB2A1	rs_HMGCS2	rs_HMGCS2	rs_C6	rs_C6
rs_CTNND2	rs_CST2	rs_S100A7	rs_S100A7	rs_ADH1C	rs_ADH1C
rs_KNDC1	rs_SLC34A2	rs_TAT	rs_TAT	rs_ADH1A	rs_ADH1A
rs_ATRNL1	rs_PSCA	rs_CYP2B7P1	rs_CYP2B7P1	rs_CLDN8	rs_CLDN8
rs_GPR98	rs_VWDE	rs_ANKRD30A	rs_ANKRD30A	rs_FOSB	rs_FOSB
rs_CLCA2	rs_LBP	rs_SERPINA6	rs_SERPINA6	rs_FABP4	rs_FABP4
rs_SERPINA5	rs_AGTR1	rs_PRAME	rs_PRAME	rs_ALDH1L1	rs_ALDH1L1
rs_PSCA	rs_C2orf54	rs_AGR3	rs_AGR3	rs_PLIN1	rs_PLIN1
rs_C8orf85	rs_SHISA2	rs_TFAP2B	rs_TFAP2B	rs_MRAP	rs_MRAP
rs_EMX1	rs_S100P	rs_KRT14	rs_KRT14	rs_HBB	rs_HBB

This table presents the top feature rankings generated by the two unsupervised machine learning models and statistical filtering of the multi-omics cancer dataset. Feature names are prefixed to indicate their data type: rs_ for single nucleotide polymorphisms (SNPs). Notably, Highly Variable Gene Selection and Spearman’s rho maintain their perfect ranking consistency, while PCA becomes inconsistent.

**Table 3:** Predictive accuracy, sensitivity, F1 score and ranking consistency of evaluated methods.

Method	Category	Ranking Consistency	Accuracy	Accuracy Rank	Sensitivity	Sensitivity Rank	F1 Score	F1 Score Rank
Linear Regression	Supervised ML	Inconsistent	0.8814 ± 0.0051	3	0.1966 ± 0.0242	3	0.2951 ± 0.0341	3
LASSO		Inconsistent	0.8769 ± 0.0057	6	0.1483 ± 0.0259	6	0.2308 ± 0.0418	6
Random Forest		Inconsistent	0.8804 ± 0.0033	4	0.2044 ± 0.1126	2	0.3014 ± 0.0283	2
XGBoost		Inconsistent	0.8814 ± 0.0038	2	0.1941 ± 0.0195	4	0.2878 ± 0.0266	4
PCA	Unsupervised ML	Inconsistent	0.8766 ± 0.0060	7	0.1104 ± 0.0347	7	0.1823 ± 0.0555	7
Highly Variable Gene Selection		Consistent	0.8862 ± 0.0025	1	0.2255 ± 0.0150	1	0.3314 ± 0.0218	1
Spearman’s rho	Statistics	Consistent	0.8797 ± 0.0037	5	0.1901 ± 0.0124	5	0.2849 ± 0.0193	5

This table summarizes the predictive performance and ranking consistency of all seven evaluated methods on the multi-omics cancer dataset, evaluated in terms of predictive accuracy, sensitivity, and F1 score. All performance metrics are reported as mean values across 10 random seeds. Highly Variable Gene Selection achieved the highest mean predictive accuracy, sensitivity, and F1 score among all methods, while also being classified as “Consistent.” Spearman’s rho was likewise classified as “Consistent” according to the predefined ranking consistency criterion. In contrast, several methods with comparable predictive performance, including Random Forest and XGBoost, were classified as “Inconsistent.”

## Data Availability

The data and source code used in this study are publicly available in Zenodo at https://zenodo.org/records/18135964 (accessed on 3 January 2026). No new data were created during this study.

## Abbreviations

The following abbreviations are used in this manuscript:

AI	Artificial Intelligence
IDC	Invasive Ductal Carcinoma
ILC	Invasive Lobular Carcinoma
LASSO	Least Absolute Shrinkage and Selection Operator
PCA	Principal Component Analysis
SNP	Single Nucleotide Polymorphism
TCGA	The Cancer Genome Atlas
XGBoost	eXtreme Gradient Boosting
